# Prognostic Impact of Percutaneous Coronary Intervention of Chronic Total Occlusion in Acute and Periprocedural Myocardial Infarction

**DOI:** 10.3390/jcm10020258

**Published:** 2021-01-12

**Authors:** Seung-Hyun Kim, Michael Behnes, Kambis Mashayekhi, Alexander Bufe, Markus Meyer-Gessner, Ibrahim El-Battrawy, Ibrahim Akin

**Affiliations:** 1 First Department of Medicine, University Medical Centre Mannheim (UMM), Faculty of Medicine Mannheim, University of Heidelberg, and DZHK (German Center for Cardiovascular Research) Partner Site Heidelberg/Mannheim, 68167 Mannheim, Germany; michael.behnes@umm.de (M.B.); Ibrahim.el-battrawy@umm.de (I.E.-B.); ibrahim.akin@umm.de (I.A.); 2 Department of Cardiology and Angiology II, University Heart Center Freiburg, 79189 Bad Krozingen, Germany; kambis.mashayekhi@universitaets-herzzentrum.de; 3 Department of Cardiology, Heart Centre Niederrhein, Helios Clinic Krefeld, 47805 Krefeld, Germany; alexander.bufe@helios-gesundheit.de; 4 University Witten/Herdecke, 58455 Witten, Germany; 5 Department of Cardiology and Intensive Care, Augusta Hospital, 40472 Düsseldorf, Germany; markus.meyer-gessner@vkkd-kliniken.de

**Keywords:** chronic total occlusion, percutaneous coronary intervention, acute myocardial infarction, periprocedural myocardial infarction

## Abstract

Coronary chronic total occlusion (CTO) has gained increasing clinical attention as the most advanced form of coronary artery disease. Prior studies already indicated a clear association of CTO with adverse clinical outcomes, especially in patients with acute myocardial infarction (AMI) and concomitant CTO of the non-infarct-related coronary artery (non-IRA). Nevertheless, the prognostic impact of percutaneous coronary intervention (PCI) of CTO in the acute setting during AMI is still controversial. Due to the complexity of the CTO lesion, CTO-PCI leads to an increased risk of complications compared to non-occlusive coronary lesions. Therefore, this review outlines the prognostic impact of CTO-PCI in patients with AMI. In addition, the prognostic impact of periprocedural myocardial infarction caused by CTO-PCI will be discussed.

## 1. Introduction

A coronary chronic total occlusion (CTO) is defined as coronary arterial occlusion without antegrade blood flow (i.e., thrombolysis in myocardial infarction (TIMI) flow 0) being present for at least 3 months. CTO has gained increasing clinical attention since it represents the most advanced form of coronary artery disease (CAD) [1,2]. In multicenter registries of consecutively evaluated patients undergoing first diagnostic coronary angiography because of symptoms of typical angina pectoris or dyspnea, CTO of major epicardial coronary arteries was present in up to one-third of patients [3,4]. Especially in patients with an acute myocardial infarction (AMI), almost 10% of patients suffer from multivessel disease (MVD) with an additional CTO [5]. In addition, up to 15% of patients with ST-segment elevation myocardial infarction (STEMI) reveal a CTO in the non-infarct-related coronary artery (non-IRA) [6].

Recent studies suggested that the presence of a concomitant CTO may significantly decrease survival in AMI patients [7,8]. A meta-analysis including more than 14,000 AMI patients demonstrated an association between the presence of a non-IRA CTO and adverse clinical outcomes in STEMI patients treated by percutaneous coronary intervention (PCI) of the infarct-related coronary artery (IRA) [9]. Patients with a non-IRA CTO had an almost three-fold increase in short-(i.e., 30 days) and long-term (i.e., 30 months) all-cause mortality compared to STEMI patients without non-IRA CTO. Moreover, the presence of CTO was associated with increased risk of major cardiovascular events (MACE), bleeding and cardiogenic shock. Another study comparing MVD STEMI patients with a CTO vs. patients without CTO demonstrated that the presence of a CTO was independently associated with increased mortality, even in MVD patients. Furthermore, the presence of a CTO was associated with increased short-(i.e., 30 days) and long-term (i.e., 5 years) all-cause mortality in patients with AMI complicated by cardiogenic shock [10].

Nevertheless, the prognostic impact of CTO-PCI in the acute setting is still controversial. In the randomized EXPLORE (Evaluating Xience and Left Ventricular Function in Percutaneous Coronary Intervention on Occlusions After ST-Elevation Myocardial Infarction) trial evaluating STEMI patients undergoing primary PCI, no impact of staged PCI of non-IRA CTO in terms of MACE was found [11]. In contrast, a recent meta-analysis including 1083 AMI patients identified that successful staged PCI of the non-IRA CTO was associated with improved prognosis at 3 years, i.e., lower rates of all-cause mortality, cardiac mortality, MACE and stroke [12]. Notably, this meta-analysis demonstrated no increased risk of repeat PCI or recurrent AMI.

Despite the potential prognostic benefits of CTO-PCI, the risk of complications in is indeed higher compared to PCI of non-CTO due to the complexity of CTO lesions [13]. The need for aggressive attempts involving dissection techniques and retrograde collateral channel crossing—especially in more complex anatomies—contributes to an increased rate of complications. Periprocedural myocardial infarction (PMI) is one of the most common complications of complex PCI. Prognostic impact of PMI is still under debate and even less data is available on the clinical impact of PMI in CTO-PCI [14]. However, the prognostic impact of PMI related to CTO-PCI is still under debate.

The following review focuses on the prognostic impact of CTO-PCI in patients with AMI. Furthermore, the benefit of a staged PCI in patients with and without cardiogenic shock will be described. Finally, the prognostic impact of PMI caused by CTO-PCI is discussed.

## 2. Pathophysiologic Role of CTO and Collateral Supply in AMI Patients

### 2.1. Pathophysiology of Coronary Collateral Connections

Coronary collateral connections (CC) are inter-arterial connections that develop already within the prenatal period and usually regress in most individuals post-partem [15,16]. However, progressive coronary atherosclerosis may induce the re-development of CC. In the presence of CTO, CC develop in all patients with CTO and viable myocardial territory in order to sustain residual oxygen supply to the myocardium of the CTO territory. CC are classified by several classification systems, especially reflecting their potential crossing profile for a planned CTO-PCI procedure (i.e., Werner and Rentrop classifications and McEntegart-, J-channel- and Huang scores) [17,18,19,20,21]. CC develop due to endothelial recruitment, arteriogenesis and angiogenesis [22]. Collateral recruitment refers to passive dilation of preformed and pre-existing CC, mainly driven by shear forces along the pressure gradient that develops when the native vessel is occluded [23,24]. In contrast, arteriogenesis is the active dilation of pre-existing CC due to vascular smooth muscle cell proliferation in order to provide enhanced blood flow to the hibernated myocardium [25]. In contrast, angiogenesis represents the migration and proliferation of capillaries regulated by vascular endothelial growth factor (VEGF) being stimulated by chronic coronary ischemia [26].

CC could develop both ipsilaterally and contralaterally. An ipsilateral CC is defined as a CC from the same main coronary artery, whereas a contralateral CC is defined as a CC from the contralateral donor coronary artery [27]. Especially in the case of CTO of the right coronary artery (RCA), ipsilateral CC were found in 30% of cases, whereas contralateral CC from the left coronary artery (LCA) were found in 92% of cases [28]. The most common ipsilateral CC reveal an epimyocardial course. In contrast, a septal intramyocardial course is more common in contralateral CC. Epimyocardial CC are mostly thinner, more fragile/vulnerable and prone to rupture, while intramyocardial septal CC reveal a straightforward and more stable course [18].

Several prior studies have already reported the protective effect of well-developed CC in CTO patients. For instance, the degree of transmural scar distal to CTO was inversely correlated with the degree of angiographic collaterals classified by the Rentrop and Werner classification [29]. Antoniucci et al. identified an association between the presence of collaterals with Rentrop grade >1 and a decreased 6-month mortality in patients with AMI undergoing primary PCI [30]. Moreover, patients with CC had lower rates of cardiogenic shock on admission. Similarly, in STEMI patients with a concomitant non-IRA CTO, the presence of well-developed collaterals (Rentrop grades 2 and 3) compared to poorly developed collaterals was associated with improved long-term survival at five years [31].

However, in contrast, coronary flow studies demonstrated that most CTO territories might be still ischemic, despite the protective effects of well-developed CC, indicating the need for CTO-PCI [32]. Werner et al. identified a high prevalence of coronary steal during hyperemia in CTO patients, which was measured by coronary flow reserve and fractional flow reserve (FFR), indicating that most patients had ischemic burden despite angiographic well-developed CC beyond the CTO [33]. In another FFR comparison study, no association between collateral grade and FFR was found, highlighting that even well-developed CC could not provide sufficient blood flow to prevent ischemia in occluded segments [34]. These findings were consistent with the result of an FFR trial including AMI patients with concomitant CTO by Lee et al., revealing a decreased myocardial FFR in all CTO patients despite the presence of well-developed CC [35]. Furthermore, Stuijzand et al. identified the ischemic burden in occluded segments irrespective of CC grade in patients with CTO, which was demonstrated by a lower myocardial blood flow and coronary flow reserve assessed by positron emission tomography (PET) [36].

In case of AMI, the protective effect of CC, even when they are well developed, may be limited due to the acute ischemia of the donor vessels, which will proceed even after primary rescue PCI [37]. These acute perfusion defects may increase the total ischemic burden that contributes consequently to an increased risk of cardiogenic shock and mortality. Sen et al. demonstrated that STEMI patients with concomitant CTO had more impaired hemodynamic parameters such as lower systolic blood pressure as well as higher heart rate and higher Killip class regardless of CC grade [38]. In a sub-analysis of the randomized EXPLORE trial including patients with STEMI and concomitant CTO, no difference in MACE, all-cause and cardiac mortality between patients with well-developed and poor-developed CC, classified by Rentrop, was shown [39].

### 2.2. Pathological Mechanism of CTO in Patients with AMI

There are several pathological mechanisms that may explain the poorer outcome in patients with AMI and concomitant non-IRA CTO. First, the worse survival in AMI patients with concomitant CTO could be explained by the so-called “double jeopardy” effect. When the previously developed CC to the ischemic area of CTO is suddenly interrupted by the acute thrombotic occlusion of the infarct-related artery (IRA), the infarcted myocardial territory refers not only to the IRA but also to the non-IRA CTO. This “double jeopardy” effect results in a larger infarct size. This pathological mechanism is supported by the HORIZONS-AMI (The Harmonizing Outcomes with Revascularization and Stents in Acute Myocardial Infarction) trial, which showed that the peak creatine phosphokinase levels (CK) tend to increase in the CTO group [6]. A study including STEMI patients with concomitant CTO also reported a trend for higher mortality in patients with well-developed retrograde CC (Rentrop grades 2 and 3) beyond the CTO originating from the occluded IRA during STEMI [31].

Secondly, microvascular ischemia and reperfusion injury are also considered as important factors to increase mortality in AMI patients with concurrent CTO [40,41,42]. After the acute occlusion of the IRA, the compensatory oxygen supply is maintained to the infarct zone via CC. However, in case of AMI with concomitant CTO, the development of CC of distal bed of IRA could be diminished when a CTO exists in one of the donor arteries, consequently inducing an increased microvascular injury. This leads to suboptimal epicardial and myocardial reperfusion in the IRA, resulting in increased volume of infarcted myocardium, reduction in ventricular function and subsequent higher mortality [43]. Claessen et al. demonstrated in AMI patients with concomitant CTO that the myocardial blush grades and ST-segment elevation resolution—as markers for epicardial and myocardial reperfusion—were decreased in CTO patients [6]. These findings are consistent with a sub-analysis from the TAPAS (the Thrombus Aspiration during Primary percutaneous coronary intervention in Acute ST elevation myocardial infarction) trial, in which patients with non-IRA CTO had reduced myocardial blush grades and ST-segment resolution [44]. Sen et al. could also reveal significantly lower rates of TIMI 2–3 flow in patients with STEMI and concomitant CTO compared to those without CTO [38].

Another important explanation for the high mortality in AMI patients with concomitant CTO is electrical instability and subsequent fatal arrhythmia. CTO could cause adverse remodeling with myocardial dilatation and dysfunction due to myocardial scaring and hibernating myocardium [32,45]. These pathological changes within the border zones may endure an abnormal electrical myocardial milieu characterized by slow inhomogeneous activation, ventricular conduction and repolarization abnormalities and consecutive cardiac death [46]. A recent study by Behnes et al. identified that the presence of CTO was an independent robust predictor of adverse prognosis in patients presenting with ventricular tachyarrhythmias (i.e., ventricular tachycardia (VT) and fibrillation (VF)). Firstly, CTO was found in 20% of patients with ventricular tachyarrhythmias, while multiple CTO were found in 22% of these patients. The presence of a native non-revascularized CTO was significantly associated with all-cause mortality at three years, notably with increased rates of the composite endpoint of early cardiac death at 24 h, recurrent ventricular tachyarrhythmias and appropriate implantable cardioverter defibrillator (ICD) therapies [47,48]. A meta-analysis by Chi et al. demonstrated that patients with an infarct-related CTO have a significantly higher risk of ventricular tachyarrhythmias, appropriate ICD therapy and increased all-cause mortality compared to those with a non-IRA CTO [49]. Another study including patients implanted with an ICD for primary prevention demonstrated that an infarct-related CTO was significantly associated with higher rates of upcoming ventricular tachyarrhythmias and cardiac mortality [50].

Furthermore, a poorer baseline ventricular function and myocardial reserve due to hibernated or scared myocardium in CTO patients may cause increased mortality in AMI. This fatal pathophysiological condition may prolong the period of myocardial stunning during acute STEMI. Prolonged myocardial stunning was shown to increase cardiac mortality in AMI patients even with a concomitant CTO [51]. A clinical trial evaluating the prognostic impact of CTO in non-IRA on left ventricular ejection fraction (LVEF) in patients with STEMI treated by PCI demonstrated that the presence of a concurrent CTO was associated not only with reduced LVEF but also with further deterioration of LVEF within one year [52].

## 3. CTO-PCI in Patients with AMI

### 3.1. CTO in STEMI

Numerous prior studies revealed the adverse outcome of a concomitant CTO in AMI patients [8,53,54]. However, the potential benefit of revascularization of CTO either by PCI or coronary artery bypass graft (CABG) in STEMI patients with or without cardiogenic shock, respectively, has rarely been investigated. There is only one randomized controlled trial available evaluating the feasibility of CTO-PCI in the setting of AMI [11]. The “EXPLORE trial” did not find any changes in LVEF (i.e., primary endpoint) and left ventricular end diastolic volume (LVEDV) assessed by cardiac magnetic resonance imaging (cMRI) at four months compared to patients without CTO-PCI, counting 304 patients. Furthermore, within the EXPLORE extension study, no differences in long-term MACE (i.e., primary endpoint) and all-cause mortality were found [55]. However, the EXPLORE trial had several relevant limitations: Firstly, CTO were not equally distributed with more frequently observed RCA—compared to left anterior descending artery (LAD) / left circumflex artery (LCX)—CTO. The study demonstrated that LAD-CTO-PCI was significantly associated with more improved LVEF values. Secondly, the staged CTO-PCI in the non-IRA was performed safely 5 ± 2 days post-primary PCI of the infarct artery, which corresponds to a time period of subacute ongoing myocardial infarction. However, this time period is characterized by ongoing inflammation, necrosis and recovery of cardiomyocytes limiting any effect on infarct size and adverse left ventricular remodeling [56,57]. Notably, the appropriate timing of staged CTO-PCI after primary PCI for AMI is still unknown. Interestingly, CTO-PCI patients had significantly less angina symptoms at one-year follow-up compared to the non-CTO-PCI group. Moreover, CTO-PCI patients with coronary multivessel disease had lower rates of MACE, suggesting that complete coronary revascularization may lead to an improvement in clinical outcomes. As assessed by cMRI, CTO-PCI in the hibernated CTO territory improved recovery of segmental wall thickening (SWT) compared to non-CTO-PCI. This finding may indicate the recovery of regional left ventricular systolic function related to CTO-PCI [58]. Interestingly, the recovery of SWT was significantly better in patients with well-developed collaterals (Rentrop grade 2–3) compared to patients with less-developed collaterals (Rentrop grade 0–1).

Further non-randomized studies by Yang and Shi demonstrated that STEMI patients with a concomitant CTO undergoing both primary and staged CTO-PCI within ten days had lower cardiac mortality and longer MACE-free survival during three-year follow-up compared to failed or conservatively treated CTO patients [59,60]. Further clinical trials indicating the consistent benefits of staged CTO-PCI in patients with STEMI are listed in Table 1 [61,62].

### 3.2. CTO in STEMI Complicated by Cardiogenic Shock

Thirty-day mortality from STEMI-associated cardiogenic shock is high at 40% to 50%, whereas in-hospital mortality rates following STEMI without cardiogenic shock are decreasing toward 3–4% [63,64]. This could be mainly explained by a higher prevalence of MVD in patients with STEMI-related cardiogenic shock, causing more myocardial ischemia and depression of LVEF at baseline. The prevalence of concomitant CTO in patients with STEMI-associated cardiogenic shock has been reported to be increased toward 24% to 29% [65]. In a sub-analysis of the Intra-aortic Balloon Pump in Cardiogenic Shock II trial (i.e., IABP-SHOCK II), STEMI patients with cardiogenic shock and concomitant CTO had higher mortality on admission at 30 days and 12 months compared to non-CTO patients [66]. Moreover, CTO patients had an increased incidence of ventricular tachyarrhythmias requiring defibrillation. The presence of CTO was also an independent predictor of cardiac mortality at 12-month follow-up. A sub-analysis from CREDO-Kyoto (the Coronary Revascularization Demonstrating Outcome study in Kyoto) registry enrolling 313 STEMI patients with cardiogenic shock underscored the prognostic importance of non-IRA CTO [67]. A size of jeopardized territory estimated by the peak CK level was higher in the CTO group, suggesting more severe hemodynamic compromise in the CTO group. This hypothesis was supported by the fact that intra-aortic balloon pumping (IABP) and/or extracorporeal membrane oxygenation (ECMO) was more frequently used in patients with a concomitant CTO. Moreover, significantly higher short- and long-term cardiac mortalities were also shown in patients with CTO. After adjusting for confounders, the presence of CTO was significantly associated with cardiac and all-cause mortality at 30-days and 5-years follow-ups. Consistent with these results, Vallabhajosyula et al. demonstrated that the presence of CTO in patients with STEMI and cardiogenic shock was associated with increased in-hospital mortality [68]. Interestingly, the impact of the number of CTO on clinical outcome was also identified [69]. Bataille et al. demonstrated that non-STEMI patients with cardiogenic shock and multivessel CTO (defined as more than two CTO lesions) survived at 30-days follow-up, whereas patients with a single CTO had a cumulative 30-days survival rate of 31.2%, and patients without a CTO had a cumulative 30-days survival rate of 55.9%. After adjustment for other independent predictors such as LVEF and renal function, the presence of CTO was still an independent predictor for 30-days mortality.

Despite the clearly adverse prognostic impact of co-existing CTO in patients with STEMI-related cardiogenic shock, the question about how to improve the survival of this high-risk group remains still unanswered. In the randomized CULPRIT-SHOCK trial (culprit-lesion-only PCI vs. multi-vessel PCI in cardiogenic shock) including 706 AMI patients with cardiogenic shock and MVD, of which almost 25% had at least a single CTO, Thiele et al. demonstrated a lower relative risk of mortality in the culprit-lesion-only PCI group than in the MVD-PCI group, including CTO-PCI [70]. Within a CULPRIT-SHOCK sub-analysis, the presence of a non-infarct-related CTO in patients with cardiogenic shock was high at 23.5% and was associated with a higher rate of death at 30 days and 12 months [71]. Currently, the CULPRIT-SHOCK trial is, however, the only randomized study evaluating prognostic differences between culprit-lesion-only PCI and MVD-PCI including CTO-PCI (i.e., overall CTO-PCI rate only 24%) in an acute unstable setting. Therefore, further randomized trials that enroll exclusively STEMI patients with cardiogenic shock and concomitant CTO should be conducted to find more appropriate treatment options for this high-risk group of patients.

### 3.3. CTO in NSTEMI

Despite advances in medical and interventional therapies, both all-cause and cardiac mortalities at a long-term follow up of ten years were estimated at 25% in patients presenting with non-ST-segment elevation myocardial infarction (NSTEMI) [72]. Similar to the prognostic impact of CTO in STEMI, CTO in patients with NSTEMI is regarded as an independent predictor of short- and long-term adverse clinical outcomes [73]. Gierlotka et al. found that patients with NSTEMI and concomitant non-IRA CTO had a significantly higher in-hospital mortality and 12-months mortality compared to patients without a CTO. It was also found that the presence of CTO was associated with higher in-hospital mortality in NSTEMI patients with cardiogenic shock [68].

Several studies underscored the advantageous prognostic impact of CTO-PCI in patients with NSTEMI. Teng et al. demonstrated a lower cardiac mortality and MACE in the successful CTO-PCI group than in the failed or non-attempted CTO-PCI group during 30-days and 2.5-years follow-ups [74]. Moreover, successful CTO-PCI was an independent protective predictor for long-term cardiac mortality. In the Korean CTO registry study, it was also demonstrated that NSTEMI patients undergoing successful CTO-PCI had a lower incidence of all-cause mortality and cardiac mortality at one year [75]. These results are consistent with the finding of the sub-analysis from the COREA-AMI registry (Cardiovascular Risk and Identification of Potential High-risk Population in Acute Myocardial Infarction) [76]. A successful CTO-PCI was associated with decreased long-term all-cause mortality and MACE in NSTEMI patients with concomitant CTO at five-years follow-up compared to failed or non-attempted CTO-PCI. However, these clinical studies indicating prognostic benefits of CTO-PCI in NSTEMI patients with concomitant CTO were not randomized, and thus, further randomized trials should be carried out to confirm its prognostic benefit.

## 4. PMI Caused by CTO-PCI

### 4.1. Definition and Pathological Mechanism of PMI Caused by CTO-PCI

The worldwide procedural success rate of CTO-PCI has impressively improved after the introduction of dedicated devices and increased operators’ clinical experience, and the threshold for performing PCI of more complex CTO has thereby been lowered [77,78]. In dedicated CTO-PCI centers, success rates of CTO-PCI have been reported of up to 95% with an acceptable risk of PCI-related complications of less than 3% [79]. Nevertheless, the higher likelihood of procedural complications of CTO-PCI compared to non-CTO-PCI still remains problematic [80,81]. One of the known complications is PMI that could be especially caused by the modern CTO approaches (such as hybrid, Euro CTO and Asia Pacific) incorporating basic to advanced PCI techniques, including antegrade and retrograde attempts with crossing of both intramyocardial and epicardial CC [82,83].

The latest revised fourth universal definition of myocardial infarction from 2018 defines an increase in highly sensitive cardiac troponin (hs-cTn) values of >5 times above the upper reference limit (URL) during the first 48 hours following PCI [plus either: (1) evidence of prolonged ischemia (≥20 min) as demonstrated by prolonged chest pain; (2) ischemic ST changes or new pathological Q waves; (3) angiographic evidence of a flow-limiting complication such as of loss of patency of a side branch, persistent slow flow or no reflow, embolization; or (4) imaging evidence of new loss of viable myocardium or new regional wall motion abnormality] as myocardial infarction associated with PCI. In contrast, the original universal definition of myocardial infarction from 2012 defined an increase in creatine kinase myocardial band (CK-MB) of 3-fold above the URL as PMI [84]. The Society for Cardiovascular Angiography and Interventions (SCAI) expert consensus committee reevaluated the prognostic impact of periprocedural biomarker elevation in 2013. A clear distinction was drawn between PMI and non-procedural myocardial infarction. With PMI, even small elevations in hs-cTn may predict increased mortality. The SCAI recommends to use an elevation in hs-cTn of >70 times above the URL or an increase in CK-MB of >10 times above the URL for definition of PMI based on the ratio of hs-cTn to CK-MB (7:1). In the presence of pathological Q waves, the SCAI expert consensus defines either an elevation in hs-cTn of >35 times above the URL or an increase in CK-MB of >5 times above the URL as PMI, which was associated with cardiovascular all-cause mortality after PCI [85,86,87,88].

The underlying pathophysiological mechanisms of PMI during CTO-PCI are known to be multifactorial. One of the determining mechanisms is, firstly, side branch occlusion [89]. CTO represents the most advanced form of CAD with long diffuse calcified lesions and a high incidence of side branch involvement, and thus, revascularization may require multiple reopening attempts with high-pressure balloons and overlapping stents, leading to compromise or occlusion of side branches. Especially in antegrade CTO procedures, side branch occlusion appeared to be the major cause of periprocedural cardiac enzyme increase [90].

Secondly, coronary microvascular obstruction may contribute to PMI [91]. Multiple and overlapping stent deployment with high-pressure balloon inflation for recanalization of CTO favors the development and perpetuation of periinterventional coronary microvascular obstruction. Due to more atherosclerotic burden, a higher incidence of microvascular embolization leading to microvascular obstruction can occur during CTO-PCI, which can be detected as small spots of myocardial necrosis by cMRI [92]. The higher number of stents used in CTO-PCI may also lead to inflammation of vessels, favoring microvascular obstruction. In addition, contrast-induced nephropathy after CTO-PCI being associated with microvascular thrombosis, platelet activation, arterial inflammation and oxidative stress could also lead to coronary microvascular obstruction [93].

Thirdly, the complex nature of CTO lesions leads to developing alternative access routes for revascularization, namely the retrograde approach, which might contribute to the occurrence of PMI [14]. The introduction of the retrograde approach has indeed helped to overcome bottlenecks of complex CTO lesion revascularization, resulting in an increased success rate of CTO-PCI. However, the incidence of PMI after CTO-PCI is more frequent in retrograde procedures compared to antegrade procedures because important CC are occluded by using the retrograde wire and microcatheter system over a long period of intervention. Interestingly, Werner et al. identified a higher incidence of hs-cTn increase in successful retrograde CTO procedures compared to unsuccessful procedures, since the mode of failure was the inability to cross the CC, and therefore, less ischemia was caused in unsuccessful CTO procedures [94].

### 4.2. Prognostic Impact of PMI Caused by CTO-PCI

Based on these pathological mechanisms, numerous clinical studies indicated that PMI after CTO–PCI may be linked to adverse clinical outcomes (Table 2) [91,95]. Kim et al. demonstrated an increased rate of MACE, cardiac and all-cause mortality in PMI patients related to CTO-PCI [96]. Similarly, another study demonstrated a higher rate of MACE in PMI patients after CTO-PCI [97]. Notably, the MACE rate increased according to increasing CK-MB levels and was highest in those patients with a 10-fold increase in CK-MB. Finally, a meta-analysis including 5.879 CTO-PCI patients from eight trials showed an increased risk of target vessel revascularization (TVR), myocardial infarction, MACE, cardiac and all-cause mortality due to PMI at minimum follow-up of one year [98]. However, whether PMI may impact mortality directly or may represent an indirect indicator reflecting progressive CAD and its complexity is still under debate [99]. This controversy is supported by data demonstrating no prognostic impact related to PMI after CTO-PCI (Table 3) [13,100,101,102]. Jaguszewski et al. demonstrated no differences in terms of MACE, cardiac death and all-cause mortality between patients with and without PMI after CTO-PCI at long-term follow-up of 10 years [102]. Interestingly, Cottens et al. demonstrated increased rates of PMI in CTO-PCI patients treated by the retrograde approach with reverse controlled antegrade and retrograde tracking (reverse CART) in line with a higher J-CTO score, whereas no difference was found regarding the rate of overall MACE [103]. Interestingly, the incidence of PMI related to CTO-PCI varies regardless of definitions of PMI. Rates of PMI related to CTO-PCI have been described between 6.8% and 11.4% according to the former definition, whereas the PMI rate has been described between 4.7% and 42% according to the new definition (Table 2 and Table 3).

The above-described controversial prognostic impact of PMI related to CTO-PCI can be explained by the difference in the two applied and changed PMI definitions within the last decade. Older studies published from 2014 to 2017 used the older definition of PMI (i.e., three-fold increase in CK-MB), whereas the newer CTO-PCI studies used the renewed PMI definition from 2018 (i.e., five-fold increase in hs-cTn), demonstrating no consisting prognostic impact related to PMI. Except the differently applied PMI definitions, all of the above-mentioned studies (Table 2 and Table 3) had comparable CTO complexities (median range of J-CTO scores: 1–3), procedural duration (median range: 80 to 140 min), rates of antegrade to retrograde approaches (median: 8% to 17%) and length of implanted stents (median: 45 to 65 mm). Again, the presence of a CTO reflects a more progressive and potentially multi-morbid disease status in the affected individual. Interestingly, current European guidelines from 2018 still recommend the use of CK-MB to assess type 5 myocardial infarction related to CABG. This recommendation is associated to the valuable area under the curve (AUC) for CK-MB, although inferior to hs-cTnI. However, it is stated that “these relationships vary depending on the nature of the procedure, the nature of cardioplegia, and the specific assay used to measure hs-cTn” as well as CK-MB [104]. CTO-PCI is known to be the most advanced and complex type of PCI, including extended procedural length and more invasive PC techniques (i.e., retrograde collateral channel crossing and blockage, CART techniques, antegrade dissection and re-entry (ADR) techniques, increasing length of implanted stents and increasing numbers of conventional angioplasty by micro- and conventional angioplasty balloons), which may potentially increase the chance of myocardial ischemia during CTO-PCI. Notably, CTO-PCI has not been differentiated within the definition of type 4a myocardial infarction [105]. Despite the prognostic impact of the older PMI definition in the setting of CTO-PCI, adapted thresholds for hs-cTnT elevation to define “novel” PMI specifically for CTO-PCI have been increased by at least 18-fold to 70-fold, where, again, significant associations with cardiac mortality at 5 years and MACE at 1 year were recently proven [14,97,106]. For instance, Koskinkas et al. demonstrated that an elevation in hs-cTn of >70 times above the URL (according to SCAI) after CTO-PCI was associated with increased one-year mortality [107]. In addition, in another study including 1.058 patients undergoing successful CTO-PCI, only an increase in CK-MB of >10 times the URL (according to SCAI) was independently associated with long-term mortality [91]. When determining thresholds for hs-cTn elevation to define “novel” PMI for CTO-PCI, it should be also considered which of the technical approaches is performed. For instance, Toma et al. retrospectively demonstrated a significant association of PMI and all-cause mortality for antegrade CTO-PCI, but not for the retrograde approach [108]. This difference may be explained by the fact that antegrade wire passage may cause more invasive antegrade dissection accompanied by side branch occlusion and distal embolization, which may, in turn, be directly associated with adverse clinical outcomes. Side branch occlusion and distal embolization are less relevant within the retrograde approach, depending on the anatomy and length of the CTO (i.e., bifurcation CTO and long CTO segments).

## 5. Conclusions

CTO represents the most advanced form of CAD and affects adverse clinical outcomes in patients with AMI due to several pathophysiological mechanisms such as the “double jeopardy” effect, microvascular ischemia, reperfusion injury, electrical instability and the limited protective role of collaterals in an acute situation. Nevertheless, the prognostic benefit of CTO-PCI, especially in the acute setting as “ad-hoc” compared to “staged PCI”, remains unclear. Although numerous clinical studies already identified the beneficial impact of staged CTO-PCI on clinical outcomes in patients with STEMI and NSTEMI, additional randomized trials need to be performed to confirm this prognostic benefit. In addition, further research is also needed to determine an adequate time interval between primary PCI and staged CTO-PCI for better clinical outcomes in patients with STEMI and NSTEMI. Additionally, further randomized clinical trials enrolling exclusively AMI patients with cardiogenic shock and concurrent CTO should be performed to better guide the clinical decision making for a staged PCI of CTO with or without additional mechanical cardiac support, such as ECMO, IABP and percutaneous ventricular assist devices. This review also outlined the controversy of the prognostic impact of PMI caused by CTO-PCI based on the changing PMI definition within the last decade. In order to solve this problem, the evidence-based thresholds of the recommended different types of cardiac biomarkers to define PMI specifically related to CTO-PCI need to be clarified and a unique definition with an adequate prognostic implication is eagerly awaited.

## Figures and Tables

**Table 1:** Clinical trials evaluating prognostic impact of a staged non-infarct-related coronary artery (IRA) chronic total occlusion percutaneous coronary intervention (CTO-PCI) in ST-segment elevation myocardial infarction (STEMI) patients.

	Elias et al. [55]	Yang et al. [59]	Shi et al. [60]	Valenti et al. [61]	Deng et al. [62]
Study design	Randomized controlled trial	Retrospective cohort study	Retrospective cohort study	Retrospective cohort study	Retrospective cohort study
Number of CTO patients	302	136	152	169	377
Duration of follow up (median)	48 months	24 months	36 months	36 months	12 months
Success rate of CTO-PCI (%)	73	64	68	78	79
Time interval between primary PCI and staged PCI (days)	5 ± 2	7–10	7–10	Up to 30	7–28
All-cause Mortality (%) (CTO-PCI vs. control)	12.9 vs. 6.2, *p* = 0.11	—	—	**3.4 vs. 15.0,** ***p* = 0.02**	**4.1 vs. 15.4,** ***p* < 0.001**
Cardiac Mortality (%) (CTO-PCI vs. control)	**6.0 vs. 1.0,** ***p* = 0.02**	**8.0 vs. 20.4,** ***p* = 0.036**	**9.0 vs. 22.9,** ***p* = 0.02**	**1.7 vs. 12,** ***p* = 0.025**	**3.6 vs. 15.0,** ***p* < 0.001**
MACE (%) (CTO-PCI vs. control)	13.5 vs. 12.3, *p* = 0.93	**21.8 vs. 38.8,** ***p* = 0.042**	**28.0 vs. 50.0,** ***p* = 0.009**	—	**4.6 vs. 19.5,** ***p* < 0.001**

CTO, chronic total occlusion; MACE, major cardiovascular events; non-IRA, non-infarct-related artery; PCI, percutaneous coronary intervention; STEMI, ST-segment elevation myocardial infarction. Bold values indicate statistically significant *p* values (*p* < 0.05), — means no information available.

**Table 2:** Studies with negative prognostic impact of CTO-PCI related to previously defined PMI.

	Lee et al. [55]	Zhang et al. [61]	Kim et al. [59]	Lo et al. [60]
Study design	Retrospective cohort study	Prospective cohort study	Prospective cohort study	Retrospective cohort study
Number of CTO patients	1058	629	337	325
Duration of follow up (median)	52 months	12 months	30 months	28 months
Definition of PMI	CK-MB ≥ 3xURL	CK-MB ≥ 3xURL	CK-MB ≥ 3xURL	CK-MB ≥ 3xURL
Rate of PMI (%)	11.4	18.3	6.8	8.6
Association of retrograde approach with PMI	**OR: 2.27,** ***p* = 0.002**	**OR: 1.35,** ***p* = 0.04**	No association found	**13.8% vs. 6.7%,** ***p* = 0.044**
All-cause mortality (PMI vs. non-PMI)	**HR: 1.96,** ***p* = 0.01**	2.6% vs. 1.0%, *p* = 0.16	**HR: 4.35,** ***p* = 0.048**	—
Cardiac Mortality (PMI vs. non-PMI)	**HR: 1.92,** ***p* = 0.04**	2.6% vs. 0.8%, *p* = 0.09	**HR: 13.3,** ***p* = 0.001**	—
MACE (PMI vs. non-PMI)	—	**12.2% vs. 4.1%,** ***p* = 0.001**	**HR: 5.2,** ***p* = 0.002**	**HR: 2.25,** ***p* = 0.006**

CK-MB, creatine kinase myocardial band; CTO, chronic total occlusion; HR, hazard ratio; MACE, major cardiovascular event; OR, odds ratio; PCI, percutaneous coronary intervention; PMI, periprocedural myocardial infarction; URL, upper reference limit. Bold values indicate statistically significant *p* values (*p* < 0.05); — means no information available.

**Table 3:** Studies without any prognostic impact of CTO-PCI related to newly defined PMI.

	Jang et al. [55]	Jaguszewski et al. [59]	Cottens et al. [60]
Study design	Retrospective cohort study	Prospective cohort study	Retrospective cohort study
Number of CTO patients	927	1110	409
Duration of follow up (median)	42 months	65 months	12 months
Definition of PMI	hs-TnI ≥ 5xURL	hs-TnI ≥ 5xURL	hs-TnT ≥ 5xURL
Rate of PMI (%)	12.7	4.7	42
Association of retrograde approach with PMI	No association found	No association found	**OR: 1.2,** ***p* = 0.04**
All-cause mortality (PMI vs. non-PMI)	HR: 1.58, *p* = 0.31	HR: 1.45, *p* = 0.33	—
Cardiac Mortality (PMI vs. non-PMI)	HR: 1.72, *p* = 0.39	HR: 2.51, *p* = 0.05	—
MACE (PMI vs. non-PMI)	HR: 1.10, *p* = 0.75	HR: 1.19, *p* = 0.49	HR: 1.37, *p* = 0.45

CK-MB, creatine kinase myocardial band; CTO, chronic total occlusion; HR, hazard ratio; hs-TnI, highly sensitive troponin I; hs-TnT, highly sensitive troponin T; MACE, major cardiovascular event; OR, odds ratio; PCI, percutaneous coronary intervention; PMI, periprocedural myocardial infarction; URL, upper reference limit. Bold values indicate statistically significant *p* values (*p* < 0.05); — means no information available.

## Data Availability

Data sharing not applicable.
